# Oridonin inhibits inflammation of epithelial cells *via* dual-targeting of CD31 Keap1 to ameliorate acute lung injury

**DOI:** 10.3389/fimmu.2023.1163397

**Published:** 2023-04-06

**Authors:** Yue Zhao, Hua Jin, Kawai Lei, Li-Ping Bai, Hudan Pan, Caiyan Wang, Xiaoming Zhu, Yanqing Tang, Zhengyang Guo, Jiye Cai, Ting Li

**Affiliations:** ^1^ State Key Laboratory of Quality Research in Chinese Medicine/Macau Institute for Applied Research in Medicine and Health, Macau University of Science and Technology, Macao, Macao SAR, China; ^2^ Department of Clinical Immunology, Institute of Clinical Laboratory Medicine, Guangdong Provincial Key Laboratory of Medical Molecular Diagnostics, Guangdong Medical University, Dongguan, China; ^3^ International Institute for Translational Chinese Medicine, Guangzhou University of Chinese Medicine, Guangzhou, Guangdong, China; ^4^ Department of Chemistry, Jinan University, Guangzhou, Guangdong, China

**Keywords:** acute lung injury, COVID-19, CD31, Keap1, oridonin, nanoparticles

## Abstract

**Introdcution:**

Acute lung injury (ALI) and acute respiratory distress syndrome (ARDS) are major causes of COVID-19 mortality. However, drug delivery to lung tissues is impeded by endothelial cell barriers, limiting the efficacy of existing treatments. A prompt and aggressive treatment strategy is therefore necessary.

**Methods:**

We assessed the ability of anti-CD31-ORI-NPs to penetrate endothelial cell barriers and specifically accumulate in lung tissues using an animal model. We also compared the efficacy of anti-CD31-ORI-NPs to that of free oridonin in ameliorating acute lung injury and evaluated the cytotoxicity of both treatments on endothelial cells.

**Results:**

Compared to free ORI, the amount of anti-CD31-ORI-NPs accumulated in lung tissues increase at least three times. Accordingly, anti-CD31-ORI-NPs improve the efficacy three times on suppressing IL-6 and TNF-a secretion, ROS production, eventually ameliorating acute lung injury in animal model. Importantly, anti-CD31-ORI-NPs significantly decrease the cytotoxicity at least two times than free oridonin on endothelial cells.

**Discussion:**

Our results from this study will not only offer a novel therapeutic strategy with high efficacy and low toxicity, but also provide the rational design of nanomaterials of a potential drug for acute lung injury therapy.

## 1 Introduction

The main mortality of hospitalized COVID-19 patients is attributed to acute viral pneumonia that causes acute lung injury (ALI) as well as the serious form, acute respiratory distress syndrome (ARDS) (1). The mortality rate from COVID-19-associated ALI/ARDS can approach 40% to 50%(2, 3).

Lungs contain 20% - 25% of the body’s entire endothelial surface which forms barrier to impede the drug delivery to the lung tissues (4). Hence, satisfactory efficacy could not be attained. Therefore, a specific treatment strategy with high efficacy and low toxicity is highly desired to treat ALI/ARDS.

Patients with ALI and ARDS exhibited substantially elevated IL-6 levels and ROS both in the blood and in the lungs (5, 6). Kelch-like ECH-associated protein 1 (Keap1) manipulates nuclear factor erythoid-2 related factor 2 (Nrf2), which is an important signaling pathway that regulates comprehensive antioxidant genes to mediate inhibitory effects on the production of ROS and proinflammatory cytokines, such as IL-6 and TNF-α (7, 8).

Oridonin (ORI) is the main active ingredient of the traditional Chinese medicine *Rabdosia rubescens* and has been approved to treat inflammatory diseases (9, 10). ORI has been reported to treat ALI by activating Nrf2 signaling (11), but it cannot effectively be accumulated in lung tissues, which limits its therapeutic effect for ALI/ARDS treatment. However, the molecular target of ORI has not been identified, and therefore, ongoing challenges, including target specificity, bioavailability and safety, restrain its development and clinical application.

Monoclonal antibody-conjugated nanoparticles (ACNPs) are a new nanodrug delivery system (DDS) that uses antibody-conjugated nanoparticles (NPs) to encapsulate drugs (12). Compared with traditional drugs, ACNPs quickly accumulate at the target site with strong specificity and few adverse events to provide a better therapeutic effect (13, 14). Some ACNPs are now reaching clinical evaluation for cancer treatment (15). Taking advantage of specific targets and controlled release, this delivery system shows potential for improving the therapeutic efficacy and reducing the toxicities of ORI in the treatment of ALI/ARDS (16, 17). However, ACNPs have not been well investigated in applications to treat the disease.

The pulmonary endothelium is a crucial orchestrator and modulator of ALI/ADRS, and serious endothelial injury in the company of intracellular virus and disturbed cell membranes is exhibited in the disease (8, 18). CD31 is predominantly localized on endothelial cells, and endothelial cells guide neutrophil migration to aggravate ALI/ARDS *via* the CD31-CD31 interaction because CD31 is also expressed on the surface of neutrophils (19). Hence, targeting CD31 could not only specifically carry drugs to lung tissues but also block the interaction between endothelial cells and neutrophils, eventually preventing neutrophil migration and infiltration to alleviate ALI/ARDS. Therefore, developing a novel targeted delivery system of ACNPS for the treatment of ALI/ARDS is promising.

In the current study, we identified Keap1 as the molecular target of ORI for the first time. We then encapsulated ORI in polyethylene glycol-polylactic acid-coglycolic acid (PEG-PLGA) to form ORI-NPs and further conjugated anti-CD31 antibodies to construct anti-CD31-ORI-NPs, a novel ACNP for the treatment of ALI/ARDS. Our results demonstrated that anti-CD31-ORI-NPs targeting CD31 selectively accumulate in the endothelial cells of lung tissues where ORI-NPs release and bind to Keap1 and in turn activate Nrf2 signaling to suppress ROS generation and IL-6 expression, eventually to treat ALI/ARDS with high efficacy and low toxicity.

## 2 Materials and methods

### 2.1 Reagents

Oridonin (> 98%) was purchased from ToYongBio (Shanghai, China). Poly(D,L-lactide-coglycolide)-b-poly(ethylene glycol)-carboxylic acid (5 k - 20 k) was purchased from Xi’an Qiyue Biology (Xi’an, Shanxi Province, China). Coumarin 6 (98%), Evans blue dye (≥ 75%), formamide, lipopolysaccharides (LPS, *Escherichia coli* O55:B5 *in vivo* and *Escherichia coli* O111:B4 *in vitro*), dichloromethane (DCM), 1-ethyl-3-(3-dimethylaminopropyl) carbodiimide hydrochloride (EDC), N-hydroxysulfosuccinimide (NHS), hexadecyltrimethylammonium bromide (HTAB, ≥ 98%), O-dianisidine dihydrochloride (O-DHC) and PVA (1%, w/w) were purchased from Sigma-Aldrich (St. Louis, MO, USA). MES hydrate (99+%) was purchased from Thermo Fisher Scientific (Waltham, MA, USA). Purified anti-mouse CD31 antibody was purchased from Biolegend (San Diego, CA, USA). Primary antibodies against VE-cadherin, Nrf2, HO-1, Keap-1, p62, and β-actin and siRNA p62 were purchased from Cell Signaling Technology (Boston, MA, USA). Phalloidin was purchased from Cytoskeleton, Inc. (Denver, CO, USA). Fluorescence-conjugated secondary antibodies (Alexa Fluor^®^ 488) were purchased from Abcam (Cambridge, UK).

### 2.2 Cell culture and animals

Primary human umbilical vein endothelial cells (HUVECs) were purchased from FuHeng Biology (Shanghai, China). HUVECs were cultured in endothelial cell medium (ECM, ScienCell Research Laboratories, Carlsbad, CA, USA) with 5% fetal bovine serum (FBS, ScienCell), containing 1% endothelial cell growth supplement (ECGS, ScienCell) and 1% penicillin/streptomycin solution (P/S, ScienCell). The cells were subcultured twice a week and incubated in a 5% CO_2_ humidified incubator at 37 °C. For all experiments, HUVECs were used for no more than eight passages.

C57BL/6 mice (8-10 weeks, 24-30 g) were purchased from Beijing Vital River Laboratory Animal Technology Co., Ltd., (Beijing, China) and cultivated in the State Key Laboratory of Quality Research in Chinese Medicine of Macau University of Science and Technology. All animal experiments were performed in accordance with Macau University of Science and Technology regulations, and the animal studies were supervised and approved by the Ethics Committee for Animal Studies at the Macau University of Science and Technology.

### 2.3 Binding affinity assay

For analysis of the binding kinetics of compounds on the recombinant proteins, biotinylation of recombinant human Keap1 protein was performed using EZ-Link™ NHS-LC-LC-Biotin according to manufacturer’s instructions (Thermo Fisher Scientific, Waltham, MA, USA). Biotinylated protein was immobilized on the streptavidin biosensor at a concentration of 6 ng μL^-1^ by incubating in phosphate-buffered saline (PBS) overnight at 4°C. ORI was dissolved in 10% DMSO-PBS solution, and different concentrations were prepared by serial dilution. ORI was incubated with biotinylated recombinant protein at different concentrations, and the binding affinity assay was analyzed by an Octet RED96 instrument (ForteBio, Fremont, CA, USA). The collected data were analyzed using custom ForteBio software.

### 2.4 Preparation of ORI-loaded PLGA-PEG nanoparticles

ORI-NPs were produced by emulsification and evaporation methods as previously described (20). Briefly, an oil phase emulsion was formed by dissolving 20 mg ORI and 60 mg PLGA or PLGA-PEG in 5 mL DCM and sonicated on ice for 3 min. Next, 20 mL of PVA (1%, w/w) was then mixed with the oil phase emulsification and ultrasonicated for an additional 3 min to form an aqueous phase emulsification. Finally, this aqueous phase emulsification was added dropwise to 60 mL of water and stirred for 6 hours to allow the DCM to evaporate resulting in the hardening of the NPs. Finally, ORI-NPs were gathered by centrifugation at 8,500 rpm for 30 min and rinsed 3 times with water. The concentrations of ORI-NPs were measured by UV-Vis Spectrophotometers (UV-2700, Shimadzu Corp., Japan) at 247 nm. Alternatively, ORI was replaced with coumarin-6 (C6), which was added to the PLGA or PLGA-PEG mixture. The concentration of NPs was determined at 460 nm with SpectraMax Paradigm Multimode Microplate Reader (Molecular Devices, San Jose, CA, USA).

### 2.5 Preparation of anti-CD31 antibody-conjugated PLGA-PEG nanoparticles

Antibody-conjugated PLGA-PEG NPs were generated *via* NHS/EDC-mediated COOH-NH2 coupling (21, 22). For activation of NPs, ORI-NPs were resuspended in 295 μL of 1 M MES (pH 5.5) under mild vortexing and allowed to equilibrate for 5 min. Then, 295 μL of EDC and NHS were instantly added to the NP solution at 100 mg mL^-1^ under mild vortexing. The activated NPs were then gathered by centrifugation for 10 min at 20,000 g to remove excess EDC and NHS. Moreover, 75 μL of MES (1 M, pH 5.5) was added to anti-CD31 antibodies or anti-IgG antibodies under mild vortexing. Then, NPs were resuspended in 50 μL of MES (50 mM, pH 5.5) using sonication and gently mixed with antibody solution. NPs and antibody solution were then mixed by vigorous vortexing for 1 hour, and the coupling reaction was terminated with 100 μL of Tris (1 M, pH 8.0). The antibody-conjugated NPs were centrifuged for 10 min at 20,000 g and resuspended in PBS. The NP concentration was determined by UV-Vis spectrophotometry.

### 2.6 Characterization of NPs

The Z-average size and zeta potential with the polydispersity index (PDI) of NPs were assessed by dynamic laser scattering (Zetasizer Nano ZS, Malvern Instruments, Malvern, UK) (23). The morphology of NPs was imagined by scanning electron microscopy (SEM, SU8020 FE-SEM, HITACHI, Ltd, Chiyoda City, Tokyo, Japan).

### 2.7 ORI entrapment efficiency

The ORI entrapment efficiency of NPs was measured by UV-Vis spectrophotometry. Briefly, the first supernatants of nanoparticles after centrifuge were collected and the amount of free drug were measured by UV-Vis spectrophotometer under 247 nm. The efficiency of drug entrapment was calculated using the following equation:


$$\text{drug entrapment (\%)=}\frac{\text{total drug - free drug}}{\text{total drug}}\times 100\%$$

### 2.8 Antibody conjugation efficiency

Antibody conjugation efficiency was evaluated by directly calculating the antibody on NPs using the Bradford protein assay (Bio-Rad, Hercules, CA). Briefly, the antibody-bound NPs were centrifuged at 8,500 rpm for 10 min, and the supernatant was saved to measure protein content. Antibody conjugation efficiency was evaluated using the following equation:


$$\text{antibody conjugation (\%) = }\frac{\text{total antibody - free antibody}}{\text{total antibody}}\times 100\%$$

### 2.9 Evaluation of ORI release from NPs *in vitro*

ORI release was estimated *in vitro* using the dialysis method as previously described (24). Briefly, dialysis bags with 1,000 Da molecular weight cut-off containing 1 mg ORI-NPs were placed in a water bath of 20 mL PBS (pH 7.4) and incubated at 37°C. At various time points, 1 mL of receiving buffer was collected and supplemented with 1 mL of PBS. The amount of ORI release from the dialysis bag into PBS was calculated by UV-Vis spectrophotometry as previously described (25).

### 2.10 Cellular uptake

HUVECs (5 × 10^4^ cells per well) were seeded in 24-well plates. Cells were cultured with anti-CD31-conjugated C6-NPs or anti-IgG-conjugated C6-NPs for the indicated time points and then rinsed 3 times in PBS to remove extra NPs. Cellular uptake of NPs was visualized using fluorescence microscopy (Olympus, Japan; *Ex* = 466 nm, *Em* = 504 nm).

### 2.11 Cell cytotoxicity assay

The cytotoxicity of NPs, free ORI, ORI-NPs or anti-CD31-ORI-NPs was evaluated using the MTT assay. Briefly, HUVECs (5 × 10^3^ per well) were cultured in 96-well plates and incubated overnight to obtain 80% confluence. After incubation with the indicated drug at various concentrations for 24 hours, 10 μL MTT solution per well (5 mg mL^-1^) was added. After another 4 hours incubation, the supernatants were aspirated out and replaced with 100 μL of DMSO. The absorbance was evaluated at 490 nm with a microplate reader. The IC_50_ value was calculated by GraphPad Prism software.


$$\text{cell survival rate }\left(\%\right)\text{ =}\frac{OD_{treatment}\;-\;OD_{blank}}{OD_{control}\;-\;OD_{blank}}\times 100\%$$

### 2.12 Murine model of LPS-induced ALI

The LPS-induced acute lung injury (ALI) model was established according to previously described methods (26). Briefly, after pretreatment with the indicated drugs by intravenous injection for 1 hour, the mice were intratracheally injected with 2.5 mg kg^-1^ LPS in saline and sacrificed in 12 hours. Tissue and blood were gathered and stored at -80°C.

### 2.13 *In vivo* NPs distribution study

LPS was intratracheally injected into C57BL/6 mice after pretreatment with the indicated drugs for 1 hour. The mice were sacrificed, and all the organs were collected after 6 hours. NP distribution was imaged using the fluorescence mode of the *In Vivo* Imaging Systems (IVIS, PerkinElmer, Inc., Waltham, MA, USA) (*Ex* = 460 nm, *Em* = 530 nm).

### 2.14 Vascular permeability assay

For determination of vascular permeability, an Evans blue dye extravasation assay was performed as previously described (27). Briefly, Evans blue (20 mg kg^-1^) was injected retro-orbitally into mice 30 min before scarification. Lungs were perfused free of blood and weighed. For quantification of the results, the lung tissues were homogenized in formamide and incubated at 56°C for over 18 hours. Homogenates were centrifuged at 14,000 rpm for 30 min, and supernatants were measured at 620 nm and 740 nm in spectrophotometer. The results are presented as microgram of EB dye per gram of lung tissue.

### 2.15 Myeloperoxidase assay

Myeloperoxidase (MPO) activity was assessed as previously described (28). Briefly, lung tissues were harvested after perfusion with sterile saline and then homogenized by a mechanical homogenizer in ice-cold 50 mM phosphate buffer (PB). Homogenates were then centrifuged at 14,000 rpm at 4°C and washed with PB. Pellets were resuspended in 500 μL of HTAB solution and freeze at -80°C for 30 min. After thawing in a 37°C water bath for 1 min, the mixtures were centrifuged at 14,000 rpm for another 10 min. Supernatants were quickly combined with PB (50 mM), 0.015% H_2_O_2_ solution and O-DHC for 5 seconds. Absorbance was assessed at 460 nm for 3 min using a DU 700 Series UV/Vis Spectrophotometer (Beckman Coulter, Brea, CA, USA). The results are presented as △OD_460_/min/g tissue.

### 2.16 Immunohistochemistry and immunocytochemistry

For histological analysis, lung tissues were fixed in 4% paraformaldehyde (PFA) and embedded in paraffin. Paraffin sections (10 μm) were then stained with hematoxylin and eosin (H&E) to analyze inflammatory cell infiltration under a microscope (Leica DM2500, Wetzlar, Germany).

For immunohistochemistry analysis, lung tissue sections (10 μm) were fixed with 4% PFA for 20 min, permeabilized using 0.1% Triton X-100, and blocked with 5% BSA. Primary antibodies (1:500) were incubated with sections at 4°C overnight. The sections were then washed in PBS with 0.1% Tween 20 solution (PBST) five times and incubated with fluorescence-conjugated secondary antibodies (Alexa Fluor^®^ 488) for 2 hours in dark. After washing with PBST and staining with DAPI, slides were mounted, and images were captured using a confocal fluorescence microscopy system (Leica TCS SP8, Wetzlar, Germany).

For immunocytochemistry analysis, HUVECs were seeded on coverslips overnight. After the treatment, the cells were stimulated with LPS. The cells were incubated for 24 hours, followed by 4% PFA fixation for 20 min. The HUVECs were permeabilized and stained with the indicated primary antibodies at 4°C overnight. The cells were incubated with fluorescence-conjugated secondary antibodies (Alexa Fluor^®^ 488) for 2 hours, followed by incubation with phalloidin and DAPI for 15 min. Finally, cytoskeleton and protein expression were visualized by a confocal fluorescence microscopy system.

### 2.17 Quantitative real-time PCR analysis

Quantitative real-time PCR was employed to determine mRNA expression. The lung total RNA was isolated with TRIzol reagent. Reverse transcription reactions were performed using Transcriptor First Strand cDNA Synthesis Kit (Roche, Inc., Basel, Switzerland). Quantitative real-time PCR analysis was conducted using real-time PCR ViiATM7 with SYBR Green master mix kit (Roche).

### 2.18 Determination of ROS production

Cell intracellular ROS was measured using DCFH-DA ROS Assay Kit according to the manufacturer’s instructions (Sigma-Aldrich, St. Louis, MO, USA). Briefly, HUVECs (3 × 10^5^ per well) were seeded in 6-well plates and were treated with compounds for 1 hour. LPS was then added to cells and incubated for 24 hours. For the assessment of ROS, after wash the cells with PBS, DCFH-DA (10 μM) was added into the cells and incubated for 30 min at 37 °C. Cells were collected and free DCFH-DA was removed. The ROS level was examined using a flow cytometer (BD FACS Aria, Franklin Lakes, NJ, USA).

For determination of ROS production in the lung, LPS-induced ALI mice models were treated with the indicated drugs for 24 hours. Lung samples were collected, ground in culture medium and isolated using a 40 μm cell strainer (Corning, Corning, NY, USA). Samples were rinsed with PBS three times and incubated with DCFH-DA at 37 °C. ROS level were analyzed using a flow cytometer.

### 2.19 Western blot and ELISAs

Western blotting was conducted according to previously described (29). Briefly, cells or tissue were lysed with RIPA lysis buffer and incubated on ice for 30 min After centrifugation, the supernatant of lysates were transferred to new tubes, and concentrations of protein were determined by BCA assay kit. Proteins were analyzed by SDS gel electrophoresis and transferred to a nitrocellulose (NC) membrane. After blocking with 5% nonfat milk or 3% BSA, the membrane was incubated with indicated primary antibodies overnight at 4 °C and secondary HRP-linked antibody for 1 hour. Blots were visualized by chemiluminescent substrate.

The expression of IL-6 in the cell supernatant was assessed using a Human Interleukin-6 (IL-6) ELISA kit (R&D Systems, Minneapolis, MN, USA) according to the manufacturer’s instructions.

### 2.20 Statistical analysis

Multiple comparisons were performed using ANOVA using GraphPad Prism software. Values of *p*< 0.05 were considered to be statistically significant. All values are the mean ± standard error of the mean (S.E.M).

## 3 Results

### 3.1 Keap1 is the molecular targets of ORI to mediate anti-inflammatory effect

**Figure 1:**
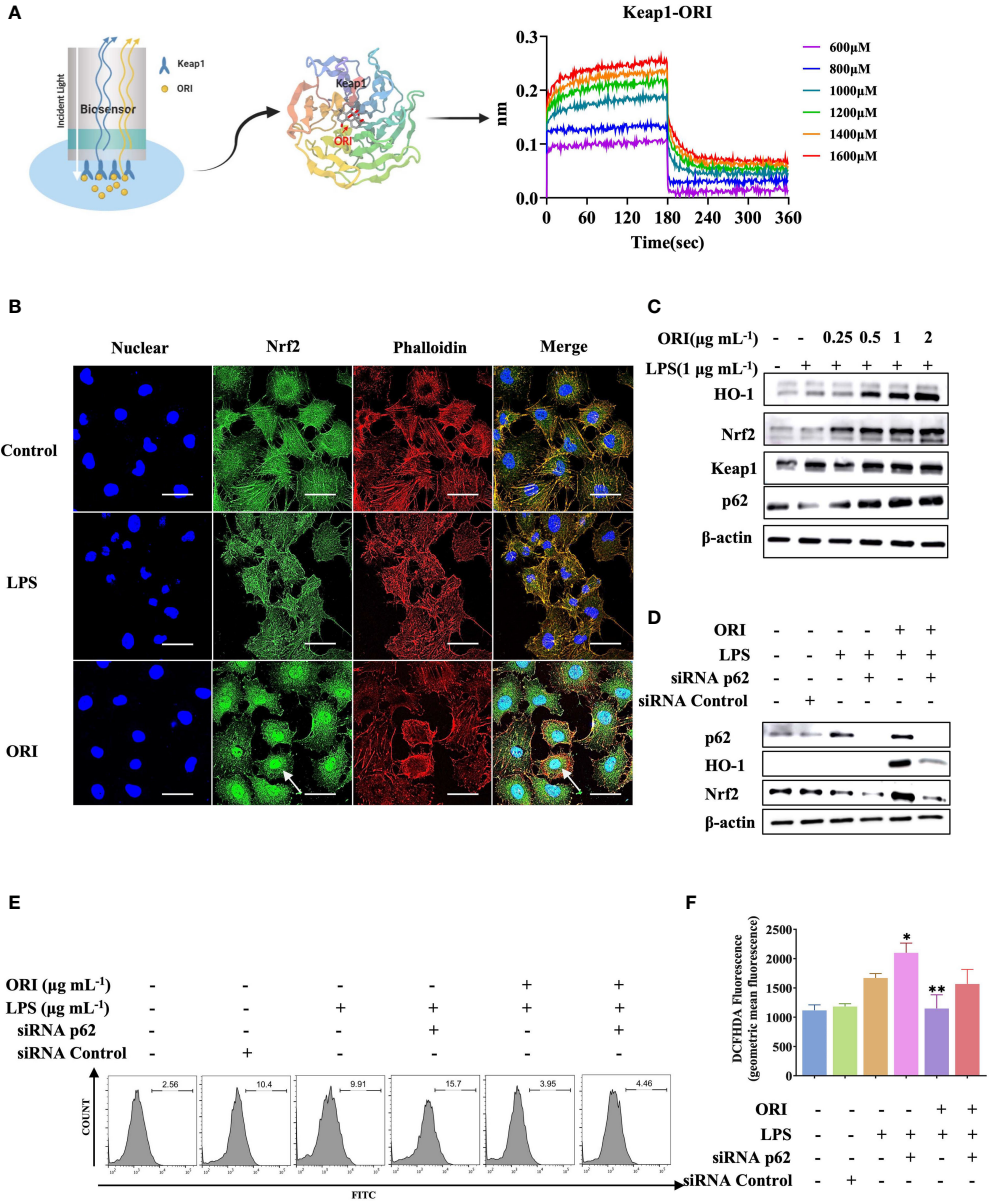
Keap1 is the molecular targets of ORI to mediate anti-inflammatory effect. **(A)** The interaction between Keap1(PDB 1U6D) and ORI was determined by binding affinity assay. **(B)** After the pretreatment with 2 μg mL^-1^ ORI, HUVECs were stimulated with 1 μg mL^-1^ LPS. Nrf2 nucleus localization was determined by immunohistochemistry. The arrow showed the Nrf2 accumulation. Bars: 50 μm. **(C)** After the pretreatment with ORI at indicated concentrations, HUVECs were then stimulated with 1 μg mL^-1^ LPS for 24 hours. Protein expression of Nrf2, Keap1, p62, HO-1 and β-actin were analyzed by Western blot analysis. **(D)** HUVECs were transiently transfected with p62 siRNA or control siRNA for 24 hours. After the pretreatment with 2 μg mL^-1^ ORI for 1 hour, the HUVECs were stimulated with 1 μg mL^-1^ LPS. The protein expression of Nrf2, p62, HO-1 and β-actin were analyzed by Western blot. **(E, F)** ROS production was measured by flow cytometry. Data were representative three independent experiments and expressed as mean ± S.E.M. *p< 0.05, **p< 0.01, compared to the LPS-stimulated group.

It was reported that ORI treated ALI and ARDS *via* activation of Nrf2 antioxidant signaling. To better understand the molecular target of ORI that mediated its antioxidant and anti-inflammatory effects, we performed a binding assay and demonstrated that Keap1 is the molecular target of ORI (**Figure 1A**). Accordingly, ORI binding to Keap1 mediated an inhibitory effect on the cytokines expression and ROS production by inducing Nrf2 nuclear localization and Nrf2-p62 signaling activation in primary HUVECs, a commonly used primary endothelial cell line for *in vitro* studies (**Figures 1B, C**; **Supplementary Figure 1**). Hence, we knocked down p62 by siRNA, and the results demonstrated that the ORI-mediated enhancement of Nrf2 accumulation was abolished in the cells (**Figure 1D**) and the inhibitory effect of ORI on ROS generation was prevented (**Figures 1E, F**). In line with the *in vitro* results, ORI inhibited ROS production, improved blood vessel permeability and suppressed proinflammatory cytokine expression both in tissue and BALF to alleviate ALI in the animal model (**Supplementary Figure 2**). These results indicated that ORI activated Nrf2-p62 signaling to inhibit inflammation of endothelial cells by binding to Keap1.

### 3.2 Preparation and characterization of anti-CD31-ORI-NPs

**Figure 2:**
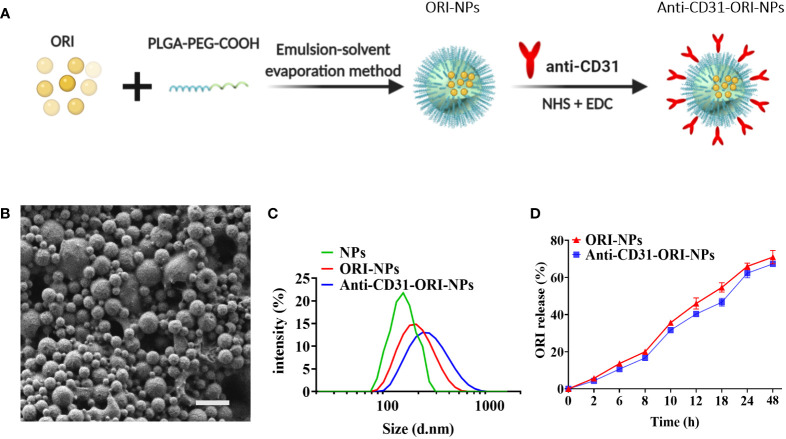
The characterization of NPs. **(A)** The preparation procedures of anti-CD31-ORI-NPs. **(B)** The morphology of NPs was observed by scanning electron microscopy. Bars:1μm. **(C)** The NPs size was detected by dynamic laser scattering. **(D)** *In vitro* release of ORI from various NPs were determined by UV spectrophotometers.

To increase the ORI bioavailability and solubility, we applied emulsion-solvent evaporation method to assemble NPs composed of ORI and PLGA (ORI-PLGA-NPs) or PLGA-PEG-block copolymers (ORI-PLGA-PEG NPs, also termed ORI-NPs) (**Figure 2A**). We found that ORI-PLGA-NPs were obviously aggregated and that the solubility was significantly reduced compared to ORI-NPs, indicating PEG decoration improve the stability and dispersibility of the ORI-PLGA-NPs (**Supplementary Figure 3A**).

Because spherical NPs were more easily internalized by cells than irregular shapes (30), we applied SEM to analyze the morphology of the ORI-NPs. As shown in **Figure 2B** and **Supplementary Figure 3B**, the ORI-NPs exhibited small, smooth and spherical particles, suggesting that ORI-NPs can more easily penetrate cells than free ORI.

It was known that CD31 is highly expressed on endothelial cells (31). To increase the targeting of ORI-NPs to the epithelia of lung tissues, we conjugated such NPs to an anti-CD31 antibody (anti-CD31-ORI-NPs) *via* covalent coupling to terminal COOH end groups on PEG. After analyzing the correlation between conjugation efficiency and antibody concentration, we found that 0.5 mg mL^-1^ was the optimal antibody conjugation concentration to achieve the highest antibody loading (**Supplementary Table 1**).

To indicate the cellular uptake efficiency and dispersion with or without anti-CD31 modification, we examined the particle size and polydispersity index (PDI) of the NPs. The results demonstrated that the size and PDI of ORI-NPs were slightly larger than those of NPs. However, there was no significant difference between anti-CD31-ORI-NPs and ORI-NPs regarding the size, PDI and absolute value of zeta potentials (**Figure 2C**; **Table 1**). We then observed the location of NPs by confocal fluorescence microscopy. The results demonstrated that the NPs accumulated in the HUVECs in a time-dependent manner and attained a peak at 6h (**Supplementary Figure 4**). Moreover, anti-CD31 antibody modification could not affect the ORI release (**Figure 2D**).

**Table 1:** Characterization of the NPs.

Nanoparticle Type	size (nm)	PDI	Zeta Potential (mV)	ORI entrapment efficiency
**NPs**	**161.6 ± 3.1**	**0.038 ± 0.002**	**-28.76 ± 0.115**	**—**
**ORI-NPs**	**192.3 ± 0.9**	**0.097 ± 0.01**	**-24.00 ± 0.964**	**71%**
**Anti-CD31-ORI-NPs**	**217.2 ± 1.34**	**0.115 ± 0.16**	**-21.7 ± 0.265**	**68%**

Values represent means ± S.E.M, n = 3.

### 3.3 Anti-CD31-ORI-NPs significantly enhanced anti-inflammatory effect of ORI *via* targeting to the endothelial cells

**Figure 3:**
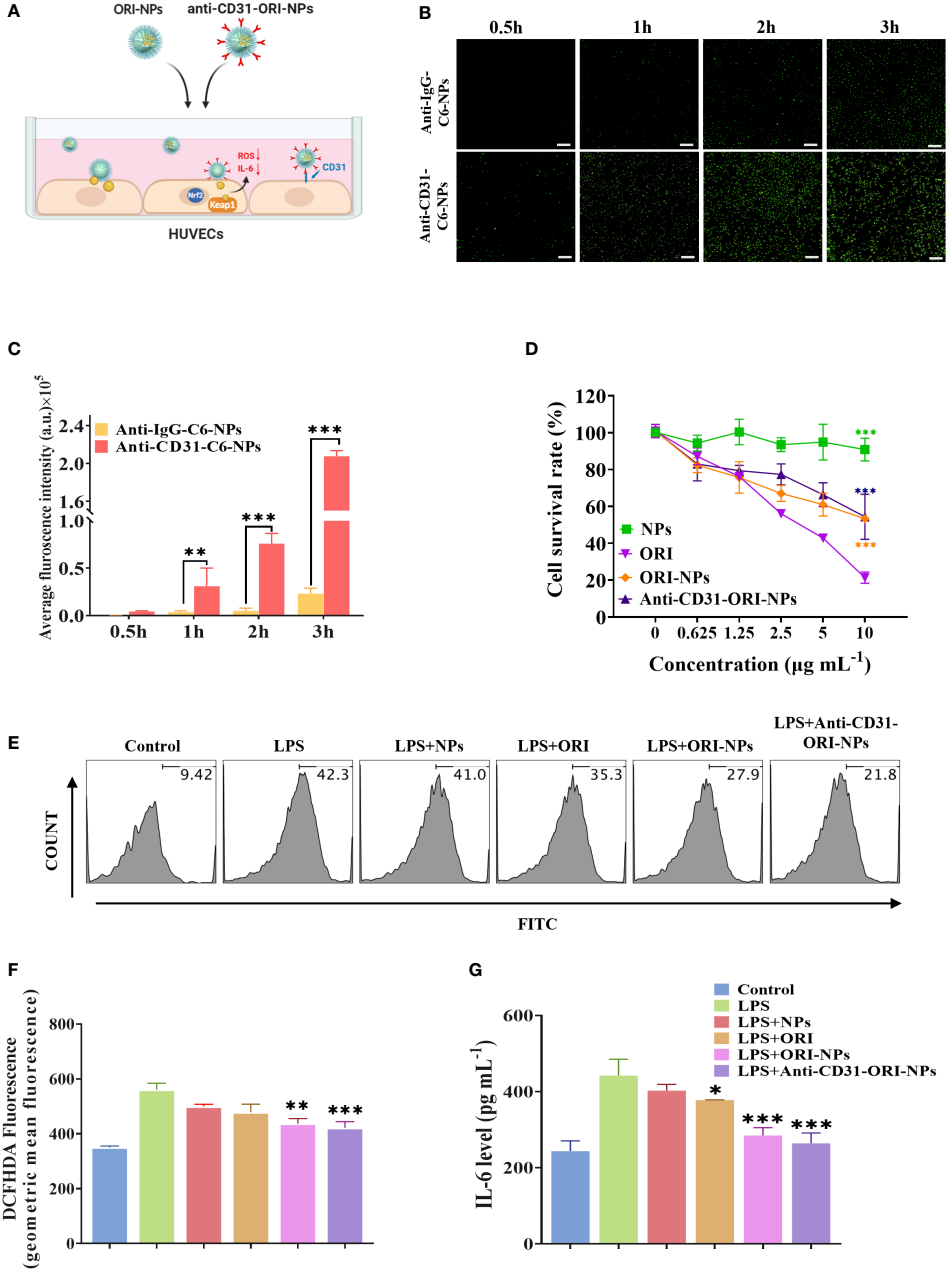
Anti-CD31-ORI-NPs enhanced anti-inflammatory effect of ORI *via* targeting to the endothelial cells **(A)** HUVECs were pretreated with 2 μg mL^-1^ blank NPs, ORI, ORI-NPs, anti-CD31-C6-NPs or anti-IgG-C6-NPs. **(B, C)** Cellular uptake of anti-CD31-C6-NPs and anti-IgG-C6-NPs in HUVECs was observed by fluorescence microscopy. The fluorescence intensity was analyzed and quantified. Bars: 1 mm. ***p< 0.001, compared to anti-IgG-C6-NPs group. **(D)** HUVECs were incubated with indicated treatments for 24 hours. The cytotoxicity was analyzed by MTT assay. *** *p*< 0.001, compared to the ORI treatment group. **(E–G)** The effects of NPs, ORI, ORI-NPs and anti-CD31-ORI-NPs on ROS and IL-6 production. HUVECs were pretreated with 2 μg mL^-1^ NPs, ORI, ORI-NPs, anti-CD31-ORI-NPs, and then stimulated with 1 μg mL^-1^ LPS. ROS and IL-6 production was evaluated by flow cytometry and ELISA, respectively. *p< 0.05, **p< 0.01 and ***p< 0.001, compared to the LPS-stimulated group. Data were representative three independent experiments and expressed as mean ± S.E.M.

To evaluate the targeting ability of anti-CD31 antibody-carrying NPs, we employed the fluorescent dye coumarin 6 (C6) instead of ORI to prepare the NPs and conjugated them to either an anti-CD31 antibody (anti-CD31-C6-NPs) or an anti-IgG antibody (anti-IgG-C6-NPs), and the NPs were then incubated with HUVECs (**Figure 3A**). The results of fluorescence microscopy clearly demonstrated that anti-CD31-C6-NPs obviously accumulated in the cells compared to anti-IgG-C6-NPs (**Figures 3B, C**), indicating that anti-CD31 modification facilitated the cellular uptake capacity of the NPs.

The toxicity of NPs is a major challenge and cannot be ignored for further clinical application. Our results demonstrated that the IC_50_ was 10 μg mL^-1^ for anti-CD31-modified and unmodified ORI-NPs, while the IC_50_ of free ORI was 4.2 μg mL^-1^ (**Figure 3D**). These results indicated that anti-CD31 conjugation effectively enhanced the binding ability of NPs to endothelial cells but decreased the cytotoxicity compared to that of ORI. Accordingly, 2 μg mL^-1^ ORI, ORI-NPs, and anti-CD31-ORI-NPs were applied in the following study to evaluate the effect on cells.

We then determined the inhibitory effect of NPs, ORI, ORI-NPs and anti-CD31-ORI-NPs on ROS production and IL-6 secretion. The results showed that anti-CD31-ORI-NPs possessed the strongest ability to reduce the intercellular ROS level and IL-6 secretion in endothelial cells compared to ORI and ORI-NPs (**Figures 3E–G**).

**Figure 4:**
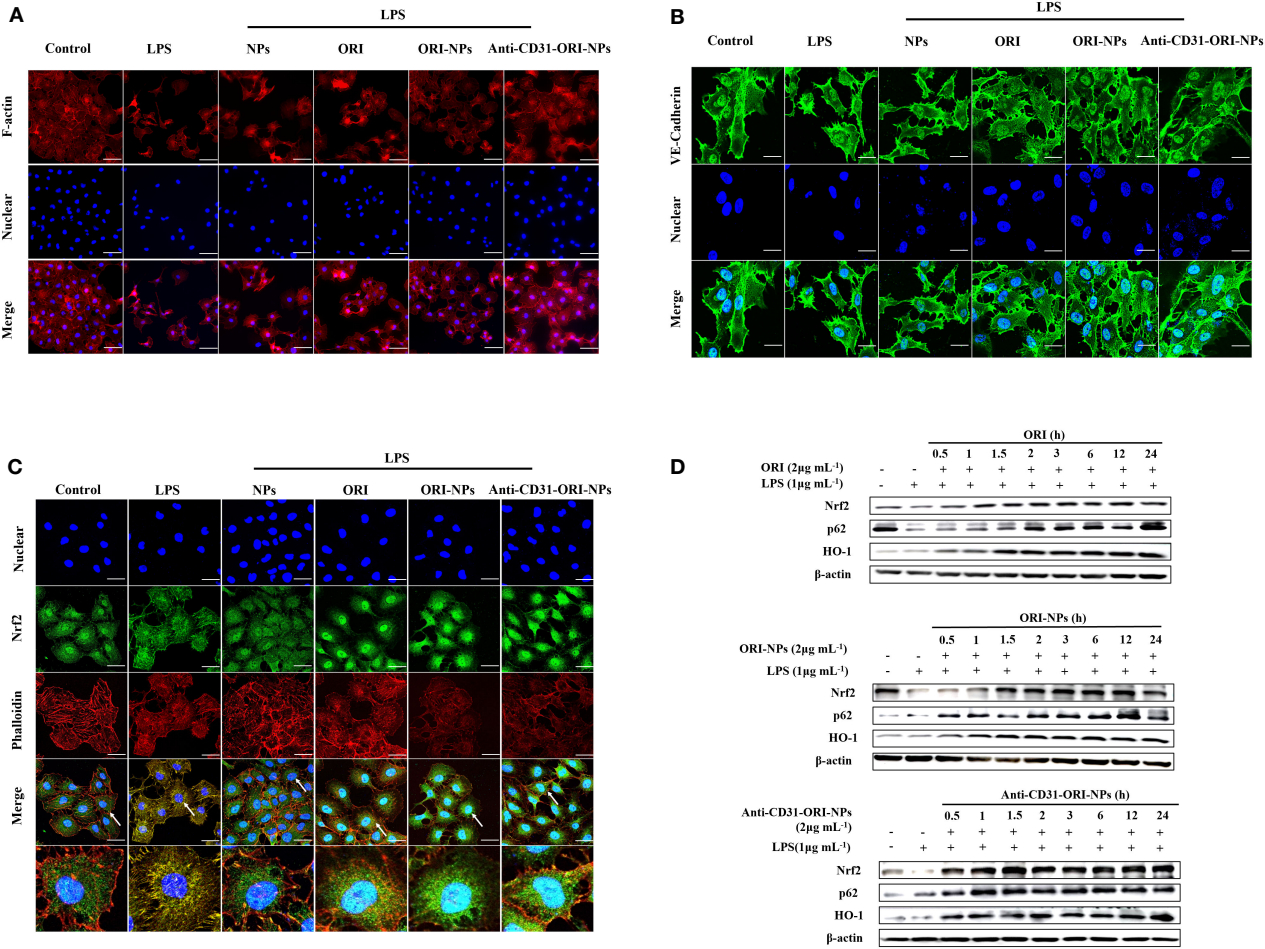
Anti-CD31-ORI-NPs recover LPS-induced endothelial injury **(A, B)** The effects of NPs, ORI, ORI-NPs and anti-CD31-ORI-NPs on cellular adhesion adjacent. After the pretreatment with 2 μg mL^-1^ NPs, ORI, ORI-NPs, anti-CD31-ORI-NPs, HUVECs were stimulated with 1 μg mL^-1^ LPS. The expression of F-actin and VE-Cadherin were analyzed by confocal fluorescence microscopy. **(C)** The effects of NPs, ORI, ORI-NPs and anti-CD31-ORI-NPs on Nrf2 nuclear localization. After the pretreatment with 2 μg mL^-1^ NPs, ORI, ORI-NPs, anti-CD31-ORI-NPs, HUVECs were stimulated with 1 μg mL^-1^ LPS. Nrf2 expression were reflected under confocal fluorescence microscopy. The arrow showed the Nrf2 accumulation. Bars: 50 μm. The nucleus was stained with DAPI (blue), and cytoskeleton was stained with phalloidin (red). F-actin, VE-Cadherin expression and Nrf2 localization were exhibited by the antibody of F-actin, VE-Cadherin or Nrf2 with fluorescence-conjugated secondary antibodies (green). **(D)** The cell extracts were prepared to determine the expression of Nrf2, p62, HO-1 and β-actin by western blot. Data were representative three independent experiments.

Filamentous actin (F-actin) and vascular endothelial cadherins (VE-cadherins) play an essential role in regulating endothelial barrier function, and the is significantly increased in endothelial cells. Under inflammatory conditions, depolymerization of F-actin and disruption of VE-cadherin adhesion lead to an increase in endothelial permeability (32). As shown in **Figures 4A, B**, we found that ORI-NPs and anti-CD31-ORI-NPs greatly restored F-actin polymerization and VE-cadherin expression, indicating that both ORI-NPs and anti-CD31-ORI-NPs have the potential to recover LPS-induced endothelial injury.

Because Keap1 is the molecular target of ORI. we then investigated whether the anti-inflammatory effects of the NPs resulted from activation of Nrf2 signaling. As our expectation, we found anti-CD31-ORI-NPs obviously increased Nrf2 accumulation and nuclear localization, p62 and HO-1 expression in the cells compared to ORI and ORI-NPs (**Figures 4C, D**).

### 3.4 Anti-CD31-ORI-NPs selectively accumulated in the lung tissues

**Figure 5:**
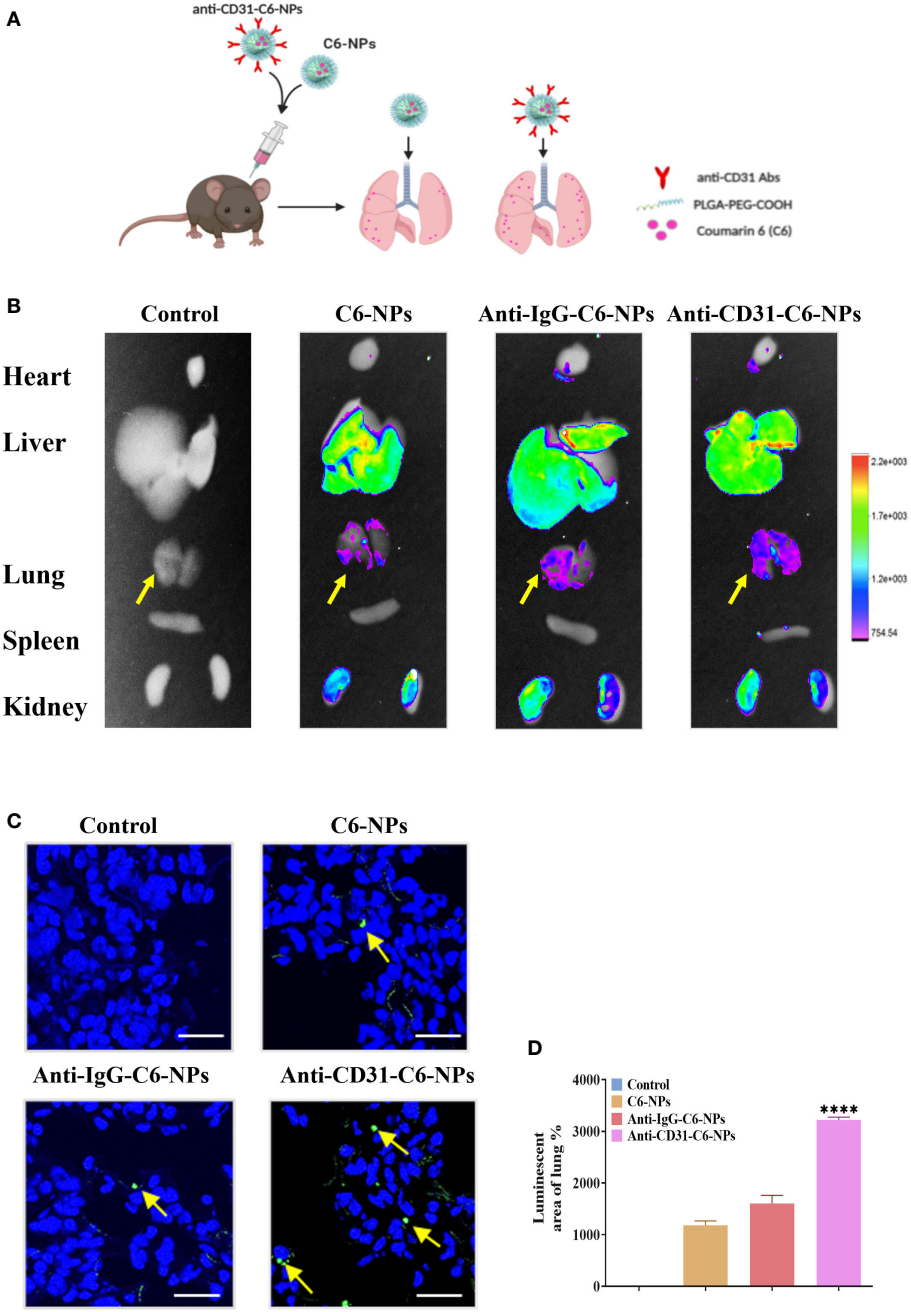
Anti-CD31-ORI-NPs selectively accumulated in the lung tissues. **(A)** C57BL/6 mice (n=3) were intravenously injected with 5 mg kg^-1^ NPs (Control), C6-NPs, anti-IgG-C6-NPs or anti-CD31-C6-NPs, respectively. The mice organs were collected for fluorescence examination by IVIS after 6 hours. **(B)** The NPs biodistribution was analyzed by *in vivo* imaging system to calculate the fluorescence intensity. **(C, D)** The fluorescence intensity of lung tissue sections was examined and quantified. Representative fluorescence image of C6 (green) distribution in lung tissue. DAPI (blue) counterstains cell nucleus. Bars: 50 μm. Data were expressed as mean ± S.E.M. ****p< 0.0001, compared to the C6-NPs group.

To further validate whether the anti-CD31 antibody conjugation could specifically accumulating the NPs in the lung tissues, we then employed an *in vivo* imaging system (IVIS) to observe the specificity and accumulation of the NPs in the different organs with or without anti-CD31 antibody modification (**Figure 5A**). Consistent with *in vitro* results, the fluorescence of anti-CD31-modified NPs was significantly and specifically concentrated in lung tissues compared with that of C6-NPs and anti-IgG-C6-NPs both at 3h and 6h treatment (**Figures 5B–D**, **Supplementary Figure 5A**). However, C6-PLGA-NP distribution in the lung tissues was obviously decreased compared to that of C6-NPs (**Supplementary Figure 5B**). These results suggested that PEG is the key component of the system to improve the solubility and delivery efficiency and targeting CD31 remarkably facilitated the NPs distributing in lung tissues.

### 3.5 Anti-CD31-ORI-NPs increases the anti-inflammatory effect to treat ALI *in vivo via* selectively accumulated in the lung tissues

**Figure 6:**
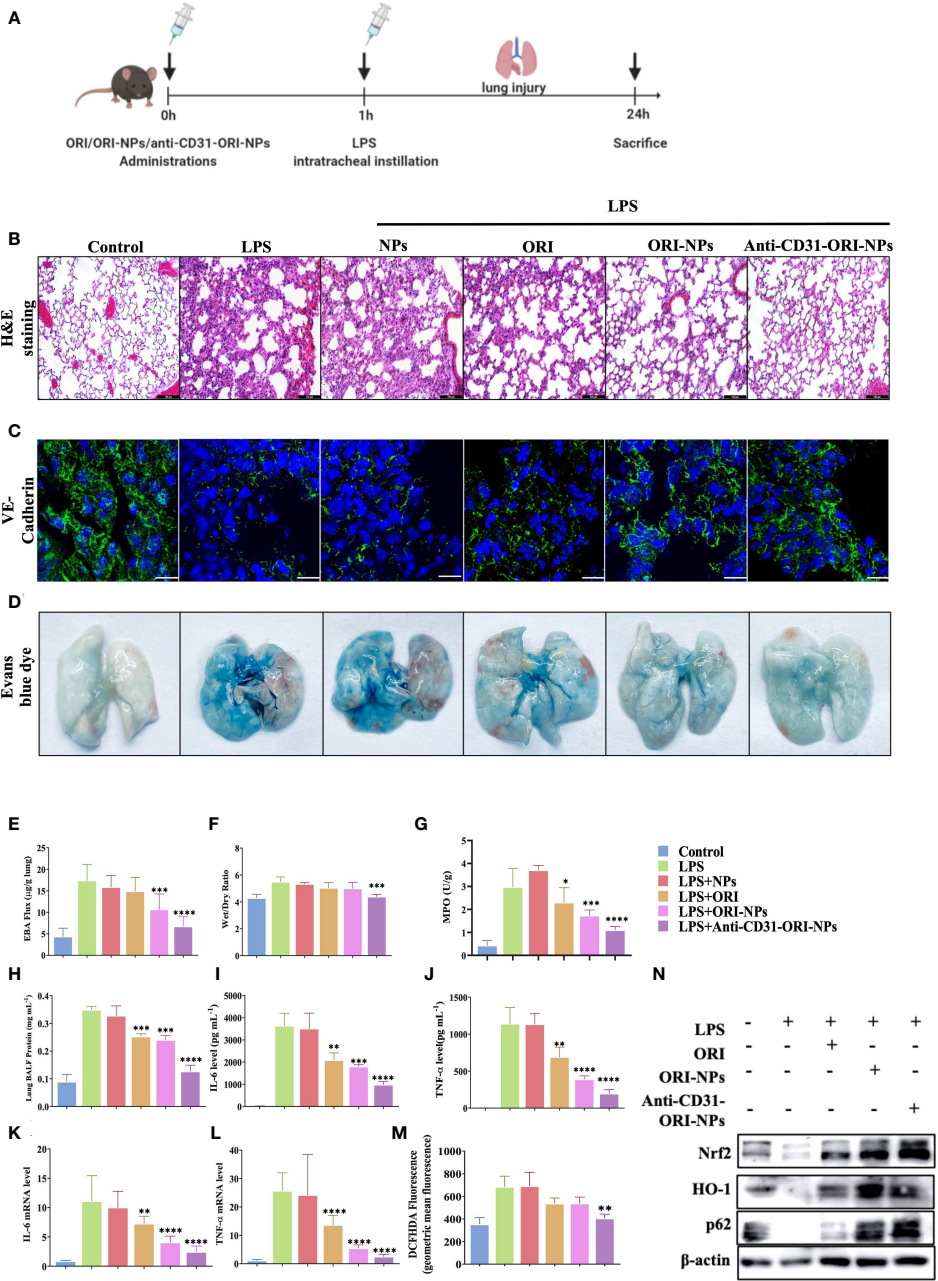
Anti-CD31-ORI-NPs increases the anti-inflammatory effect to treat ALI *in vivo via* selectively accumulated in the lung tissues. **(A)** C57BL/6 mice (n=10) were intravenously injected 5 mg kg^-1^ NPs, ORI, ORI-NPs and anti-CD31-ORI-NPs for 1 hour, respectively. Mice were then intratracheally injected with 2.5 mg kg^-1^ LPS. Mice were sacrificed and the tissues were collected. **(B)** Representative histological section of the lungs was stained with hematoxylin and eosin. Bars: 100 μm. Magnification × 200. **(C)** The sections of lung tissues were prepared to determine the expression level of VE-cadherin with fluorescence-conjugated secondary antibodies (green). DAPI (blue) counterstains cell nucleus. Bars: 50 μm. **(D, E)** The pulmonary vascular permeability of lung tissues was evaluated and quantified by Evans blue extravasation assay. **(F)** The wet-to-dry ratios of lungs derived from different treatment groups were calculated. **(G)** The neutrophil migration and infiltration were determined by measuring MPO activity. **(H)**The protein concentration in BALF was quantified using Bradford protein assay. **(I, J)** The secretion of IL-6 and TNF-α in BALF were determined by ELISA. **(K, L)** The mRNA expression of IL-6 and TNF-α in lungs was quantified by QRT-PCR. **(M)** ROS production in the lung tissues was analyzed by flow cytometry. **(N)** The expression of Nrf2, p62, HO-1, and β-actin in the lung tissues with indicated treatments were examined by western blot. Data were expressed as mean ± S.E.M. *p< 0.05, **p< 0.01, ***p< 0.001 and ****p< 0.0001, compared to the LPS-stimulated group.

Since anti-CD31-ORI-NPs mainly accumulated in lung tissues by targeting CD31, we determined whether intensive treatment could be provided in ALI mouse model (**Figure 6A**). In coincided with the results, ORI, ORI-NPs and anti-CD31-ORI-NPs inhibited neutrophil infiltration and increased VE-cadherin expression level in lung sections derived from the mice with ALI. Among of the treatments, anti-CD31-ORI-NPs showed the strongest ability to inhibit the inflammation and restore the level of ZO-1, Occludin and VE-Cadherin expression (**Figures 6B, C**; **Supplementary Figure 6A**).

Under physiological conditions, the endothelium is impermeable to albumin. In acute lung injury, vascular endothelial cells were damaged resulting in the increase of capillary permeability, and fluids from capillaries leak into the interstitial space and the alveoli, causing pulmonary edema and filling with protein-rich edema fluid. Evans blue, which can bind serum albumin, was used to test blood vessel permeability.(33) In the study, we found that anti-CD31-ORI-NPs remarkably recovered the permeability in the lung tissues compared to unmodified ORI-NPs, although ORI-NPs demonstrated obvious restoration of the permeability compared to ORI (**Figures 6D, E**). Consistent with the results, the wet/dry ratio from the lung tissues with the indicated treatments showed the same trend (**Figure 6F**). Neutrophil migration and infiltration are usually assessed by myeloperoxidase (MPO) assays and associated with NET formation, one of the features of ALI/ARDS in COVID-19 patients. Our results demonstrated that MPO activity could be significantly attenuated by anti-CD31-ORI-NPs, which was better than ORI or ORI-NPs (**Figure 6G**).

To further validate that the effect of anti-CD31-NPs-ORI on the mice with ALI resulted from inhibition of ROS release and cytokines production, we examined ROS generation, mRNA expression and secretion of IL-6 and TNF-α. The results showed that ROS levels and IL-6 expression were suppressed in the lung tissues of the treated groups compared to the LPS-stimulated group. TNF-α expression was also obviously suppressed by ORI-NPs and anti-CD31-ORI-NPs. Remarkably, the anti-CD31-ORI-NP treatment was the best among the indicated treatments (**Figures 6H–M**; **Supplementary Figure 6B**). H&E results demonstrated that the NPs have no influence on the morphology of the kidney and liver, indicating the NPs did not exhibit significant toxicity on the tissues **(** **Supplementary Figure 7**).

These results demonstrated that anti-CD31-ORI-NPs improved the efficacy at least three times compared with free ORI to ameliorate ALI. In line with the beneficial effect in the mice with ALI and the *in vitro* results, anti-CD31-ORI-NPs were the most effective at enhancing the p62-Nrf2-HO-1 axis (**Figure 6N**). These results suggested that anti-CD31-ORI-NPs enhanced the accumulation of NPs in the lung and in turn suppressed ROS production, inhibited IL-6 mRNA expression and restored endothelial injury to ameliorate ALI *via* activation of the Nrf2-p62 feedback loop.

## 4 Discussion

Nearly 50% of non-survivors of COVID-19 patients experienced secondary bacterial infections and induced ALI and ARDS causing the mortality of patients (34), but so far, there is no effective drug in clinical application. Compared to the patients with influenza-associated ARDS, the patients with COVID-19 showed severe endothelial injury associated with the presence of intracellular virus and disrupted cell membranes, which induced neutrophil infiltration in pulmonary capillaries and migrated into the alveolar spaces, leading to neutrophilic mucositis and NETs (35, 36). Neutrophil infiltration generates production of ROS, which contributes to COVID-19 disease severity *via* induction of tissue damage and thrombosis. Furthermore, it was reported that SARS-CoV-2-triggered NETs mediate COVID-19 pathology and NET formation requires ROS (37, 38). These studies indicated that suppression of ROS production and neutrophil activation are important strategies to treat ARDS and ALI associated with COVID-19.

Despite understanding of the pathogenesis of ALI, therapeutic strategies for this disease are still limited. Due to the pathogenesis role of ROS in ALI, it is now widely recognized that activation of Keap1-Nrf2-p62 axis is a key mechanism. Because Keap1 possesses highly reactive cysteine and modification of cysteine thiols of Keap1 most likely changes the structure of the Nrf2/Keap1 complex and activation of Nrf2, Keap1 is considered as a molecular target for the prevention and treatment of many diseases associated with oxidant stress. In recent years, increasing natural products have been used to attenuate the symptoms of ALI/ARDS models (39–41). However, the efficiency and safety of is still a challenge for clinical application of those compounds. Previous studies have shown that ORI possessed potent anti-inflammatory activates (9, 42, 43), while the molecular target of ORI has not been well elucidated. Our results revealed that ORI directly bound to Keap1 to activate p62-Nrf2 feedback loop and inhibited ROS production to attenuated ALI symptoms. Importantly, ORI could significantly suppress mRNA expression and secretion of IL-6, which is one of crucial cytokines contributing to the “cytokine storm” of the patients with COVID-19. However, the clinical application of ORI is restrained by its tolerance and efficacy, although it has been approved in China to treat inflammatory diseases.

Compared with traditional drugs, nanomedicines can quickly accumulate at the target site at high concentration with strong specificity and low side effects. NPs loading a variety of drugs and biomolecules have been applied to treat ALI due to specific target, controlled release and well tolerance (16). However, intracellular proteins are easily adsorbed on NPs, forming the so-called protein corona (PC), which may greatly change NP’s surface charge, affect their dispersion and ability to recognize targets (44), and then remarkably reduce the efficacy of the drugs carried by the NPs. NPs modified by PEG can change the surface charge and prevent the formation of protein crowns and the degradation of NPs (45). However, the delivery system of NPs-PEG could not effectively act on the injured lung. Therefore, development of delivery system of drugs directly to the desired lung site is under spot light.

ACNPs is a newly drug delivery system taking advantages of ADC and nanotechnology by using antibodies conjugated NPs encapsulating drugs. Compared with ADC and NPs, ACNPs overcome pharmacokinetic limitations and ensure the drug delivered to the site in the required amount to provide a better therapeutic effects (12). It was reported that ICAM-1 modified drug can alleviate pulmonary inflammation by directly delivered to lung (46). Unlike ICAM-1 localized in the luminal membrane (47), CD31 is localized predominantly in the neutrophil and interendothelial borders, and the lung tissues are rich in capillaries and endothelial cells, which have a large surface area for receiving targeted therapy. Notably, endothelial cells interact with neutrophil to mediate neutrophil migration and infiltration *via* CD31-CD31 (48). Therefore, anti-CD31 antibodies not only specifically target to the endothelial of lung tissues but also prevent neutrophil migration and infiltration to form NETs in ARDS and ALI patients. Hence then, CD31 is an ideal target to increase the efficacy of the encapsulated drug to treat ARDS and ALI.

**Figure 7:**
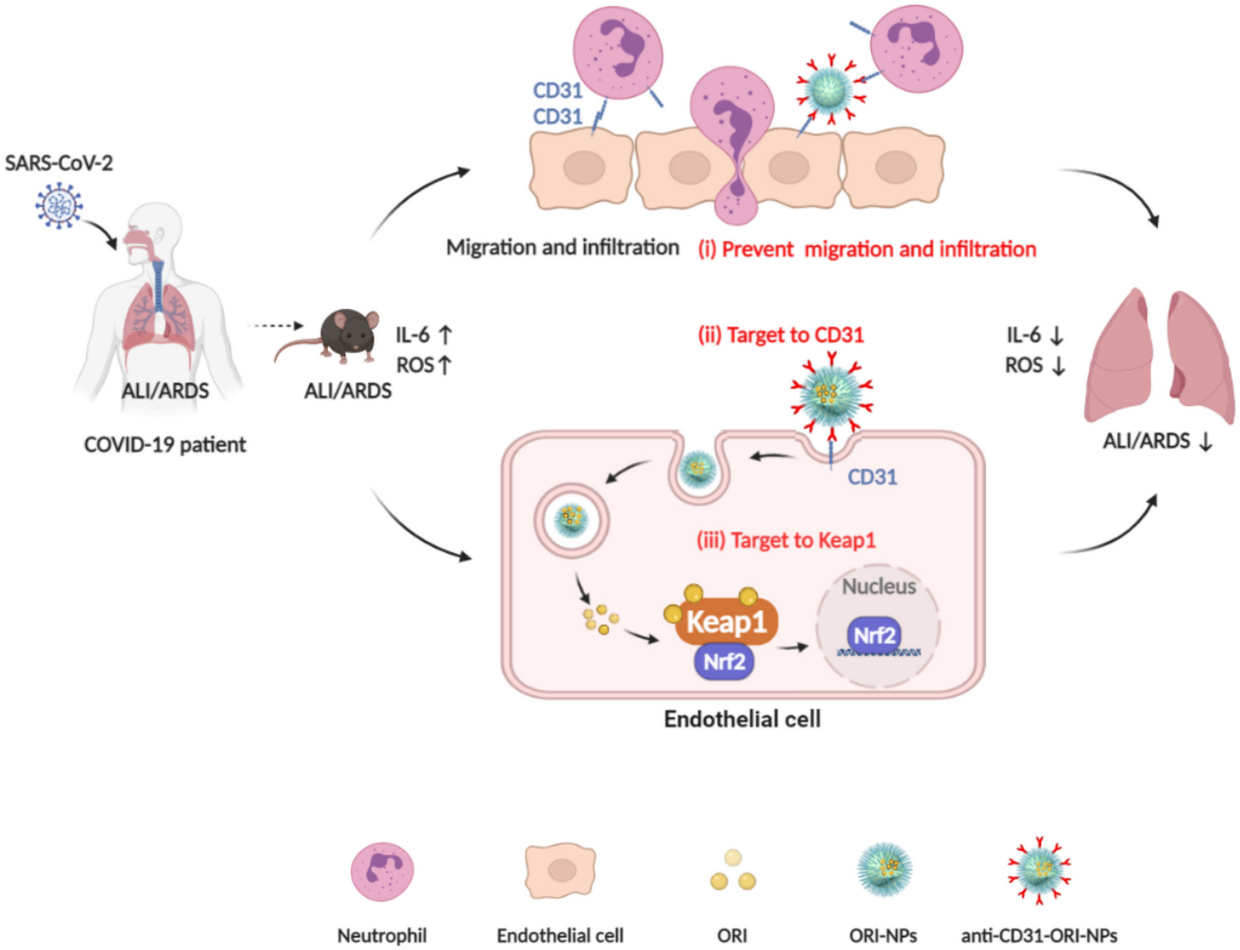
The mechanism of anti-CD31-ORI-NPs to ameliorate ALI/ARDS. (i), anti-CD31-ORI-NPs prevent neutrophil migration and infiltration *via* interruption of CD31-CD31 bond. (ii), anti-CD31-NPs specifically target to endothelial cell *via* CD31 for ORI release to inhibit ROS and IL-6 expression *via* (iii), binding to Keap1.

In the present study, we prepared a novel delivery system of anti-CD31-conjugated oridonin-PLGA-PEG nanoparticles (anti-CD31-ORI-PLGA-PEG NPs, also termed anti-CD31-ORI-NPs). The system showed highly efficient treatment ALI/ARDS by exploiting the advantages of these materials, including specific targeting to endothelial cells *via* CD31, the accurate identification of its molecular target Keap1 by the anti-inflammatory drug ORI, and the controlled release and few side effects of PLGA-PEG NPs (**Figure 7**).

By immunotargeting CD31, we specifically delivered ORI to endothelial cells, where ORI was released and bound to Keap1 to inhibit ROS and IL-6 expression and suppress ALI and ARDS. However, endothelial cells mediate neutrophil transmigration *via* CD31-CD31 under inflammatory conditions and in turn aggravate ALI and ARDS. By immunotargeting CD31, anti-CD31-ORI-NPs interfered with the interaction between endothelial cells and neutrophils to attenuate neutrophil transmigration and infiltration, eventually ameliorating ALI and ARDS.

## 5 Conclusion

In conclusion, our study provides intensive evidence *in vitro* and *in vivo* to demonstrate that anti-CD31-ORI-NPs targeting endothelial cells in lung tissues not only enhanced the anti-inflammatory and antioxidant effects of ORI but also prevented neutrophil migration and infiltration to alleviate ALI/ARDS with low cytotoxicity. This high efficacy and low toxicity nanomedicine with dual targets may pave a new way for the development of approved anti-inflammatory drugs to combat ALI and ARDS in COVID-19 patients.

## Data availability statement

The original contributions presented in the study are included in the article/**Supplementary Material**. Further inquiries can be directed to the corresponding author.

## Ethics statement

The animal study was reviewed and approved by Macau University of Science and Technology.

## Author contributions

TL conceived the study and supervised experiments. TL and YZ wrote the manuscript. YZ and HJ, performed experiments, analyzed data, and assisted in manuscript writing. KL, ZG, CW and YT performed experiments. XZ and JC assisted in manuscript writing. L-PB and HP provided the financial support. All authors contributed to the article and approved the submitted version.
