# Supplementary material for: Oridonin inhibits inflammation of epithelial cells via dual-targeting of CD31 Keap1 to ameliorate acute lung injury
Source: Front Immunol. 2023 Apr 6;14:1163397. doi: 10.3389/fimmu.2023.1163397 (PMC10116055; doi:10.3389/fimmu.2023.1163397)
Supplement: Supplementary file 1 [file DataSheet_1.pdf]

Supplementary Material

Oridonin inhibits inflammation of epithelial cells via dual-targeting of CD31 and Keap1 to ameliorate acute lung injury

Yue Zhao, Hua Jin, Kawai Lei, Li-Ping Bai, Hudan Pan, Caiyan Wang, Xiaoming Zhu, Yanqing Tang, Zhengyang Guo, Jiye Cai, Ting Li*

*Correspondence:
Ting Li
tli@must.edu.mo

This supporting information includes **Supplementary Table S1** and **Supplementary Figure S1 to S7**

Supplementary Table 1 Antibody conjugation efficiency

Concentration of anti-CD31 Ab (mg mL^{-1})	Ab conjugation efficiency	Ab loading (μg Ab/mg NPs)
**0.25**	**58.7 ± 2.1%**	**36.68 ± 1.32**
**0.5**	**42.0 ± 3.2%**	**52.55 ± 4.12**
**1**	**20.8 ± 1.5%**	**51.97 ± 3.93**

Values represent means ± S.E.M, *n*= 3.

Supplementary Figure. 1

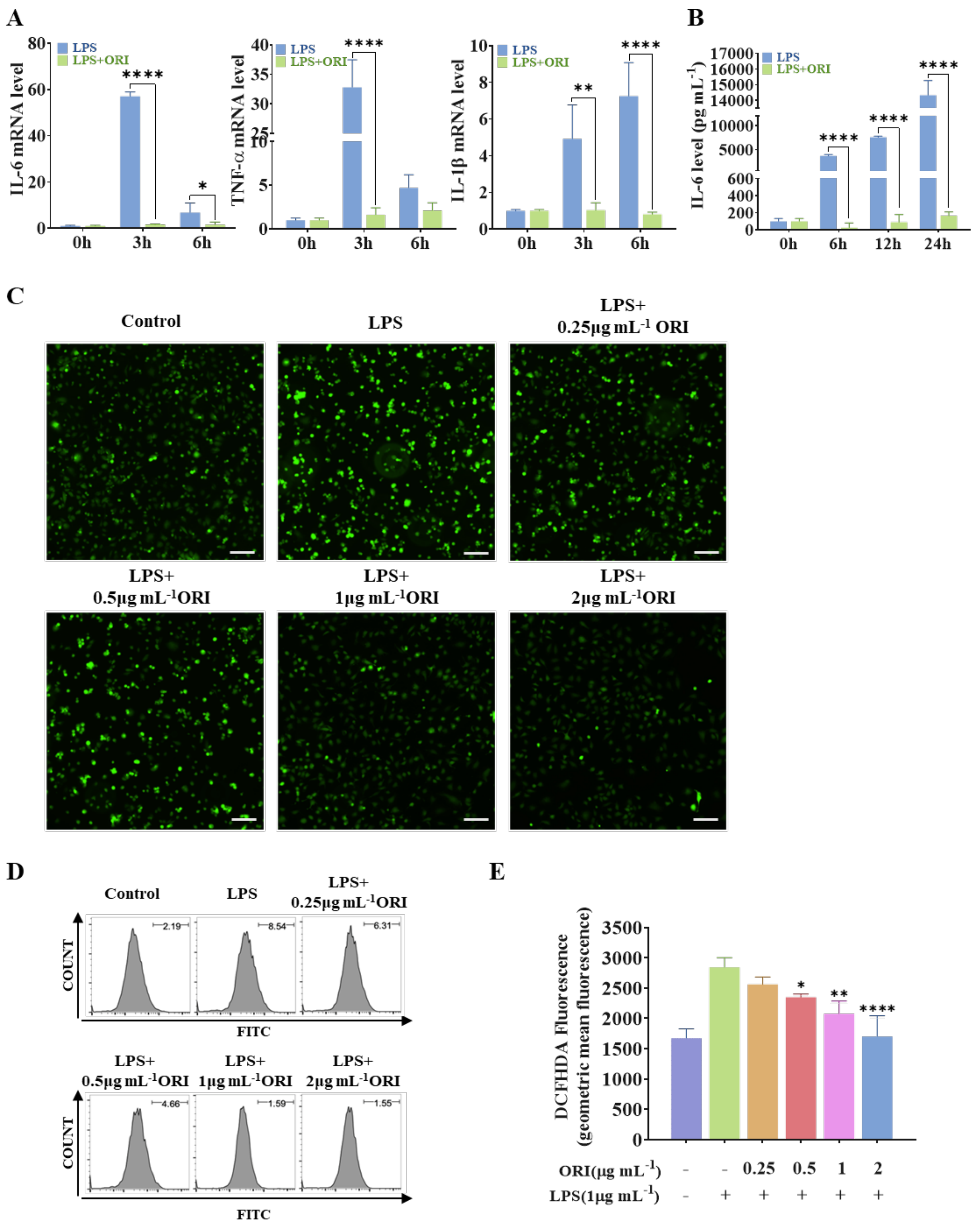

Supplementary Figure 1 ORI suppressed inflammation of endothelial cells. After the pretreatment with 2 μg mL^{-1} ORI for 1 hour, HUVECs were stimulated with 1 μg mL^{-1} LPS for indicated time. (A-B) The mRNA expression and secretion of IL-6, TNF-α and IL-1β were determined by QRT-PCR and ELISA, respectively. (C-E) ROS production was measured by fluorescence microscopy and flow cytometry. Bars: 200 μm. Data were representative three independent experiments and expressed as mean ± S.E.M. *$p < 0.05$, **$p < 0.01$, ***$p < 0.001$ and ****$p < 0.0001$, compared to the LPS-stimulated group.

Supplementary Figure. 2

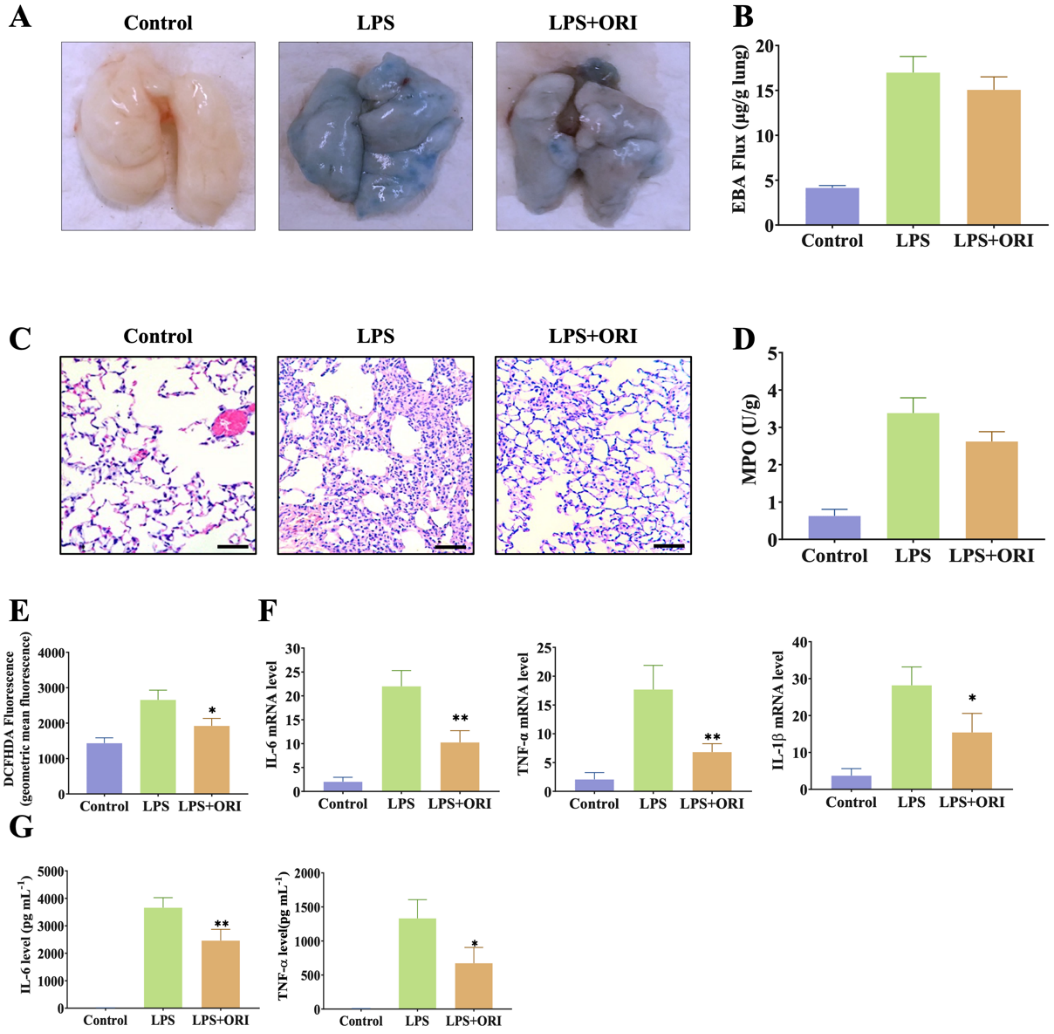

Supplementary Figure 2 ORI attenuated ALI animal model. C57BL/6 mice (n=10) were pretreated with 5 mg kg^{-1} ORI for 1 hour following intratracheally injected with 2.5 mg kg^{-1} LPS, respectively. The mice were sacrificed, and the lung tissues were isolated. (A-B) the pulmonary vascular permeability was evaluated and quantified by Evans blue extravasation assay. (C) Representative histological section of the lungs was stained by hematoxylin and eosin. Bars: 100 μm. Magnification × 200. (D) Neutrophil migration and infiltration were assessed by myeloperoxidase (MPO). (E) ROS production in the lung tissues was analyzed by flow cytometry. (F) mRNA expression of IL-6, TNF-α and IL-1β in the lung tissues was determined by QRT-PCR. (G) The secretion of IL-6 and TNF-α in BALF were determined by ELISA Data were representative three independent experiments and expressed as mean ± S.E.M. *$p < 0.05$, **$p < 0.01$, compared to the LPS-stimulated group.

Supplementary Figure. 3

A

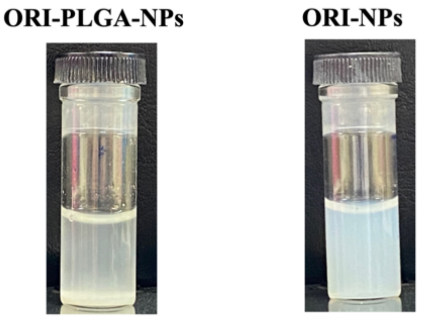

B

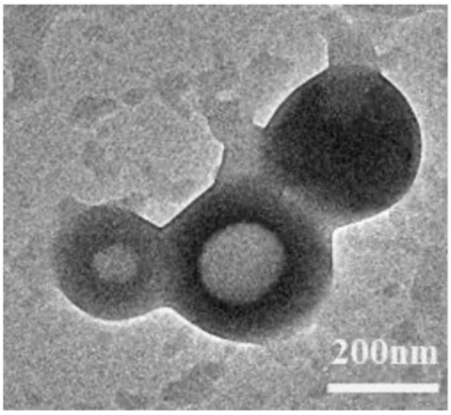

Supplementary Figure 3 (A) ORI-PLGA-NPs (left) and ORI-NPs (right) were dissolved in the PBS.

(B) The morphology of NPs was observed by transmission electron microscopy. Bars :200 nm.

Supplementary Figure. 4

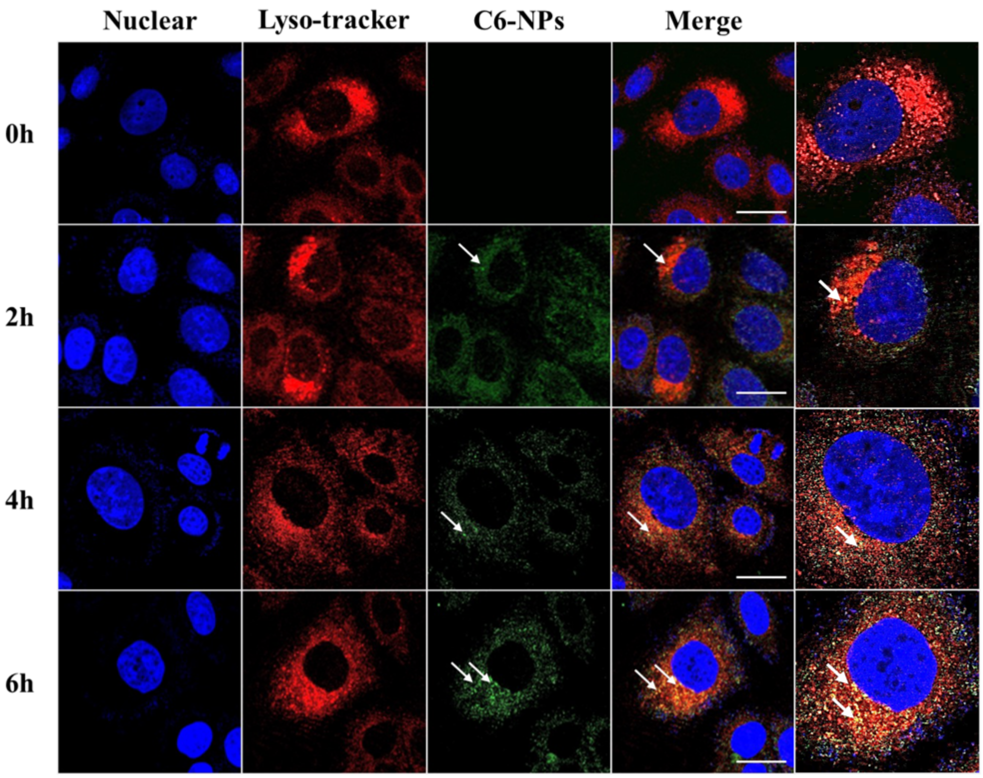

Supplementary Figure. 4 Co-localization of C6-NPs and lysosomes were observed by confocal fluorescence microscopy in HUVECs. HUVECs were incubated with C6-NPs (Green) for different time (0 h, 2 h, 4 h and 6 h), followed stained with Lyso-tracker (Red) for 1hours and DAPI (Blue) for 5 min, Bars: 25μm.

Supplementary Figure. 5

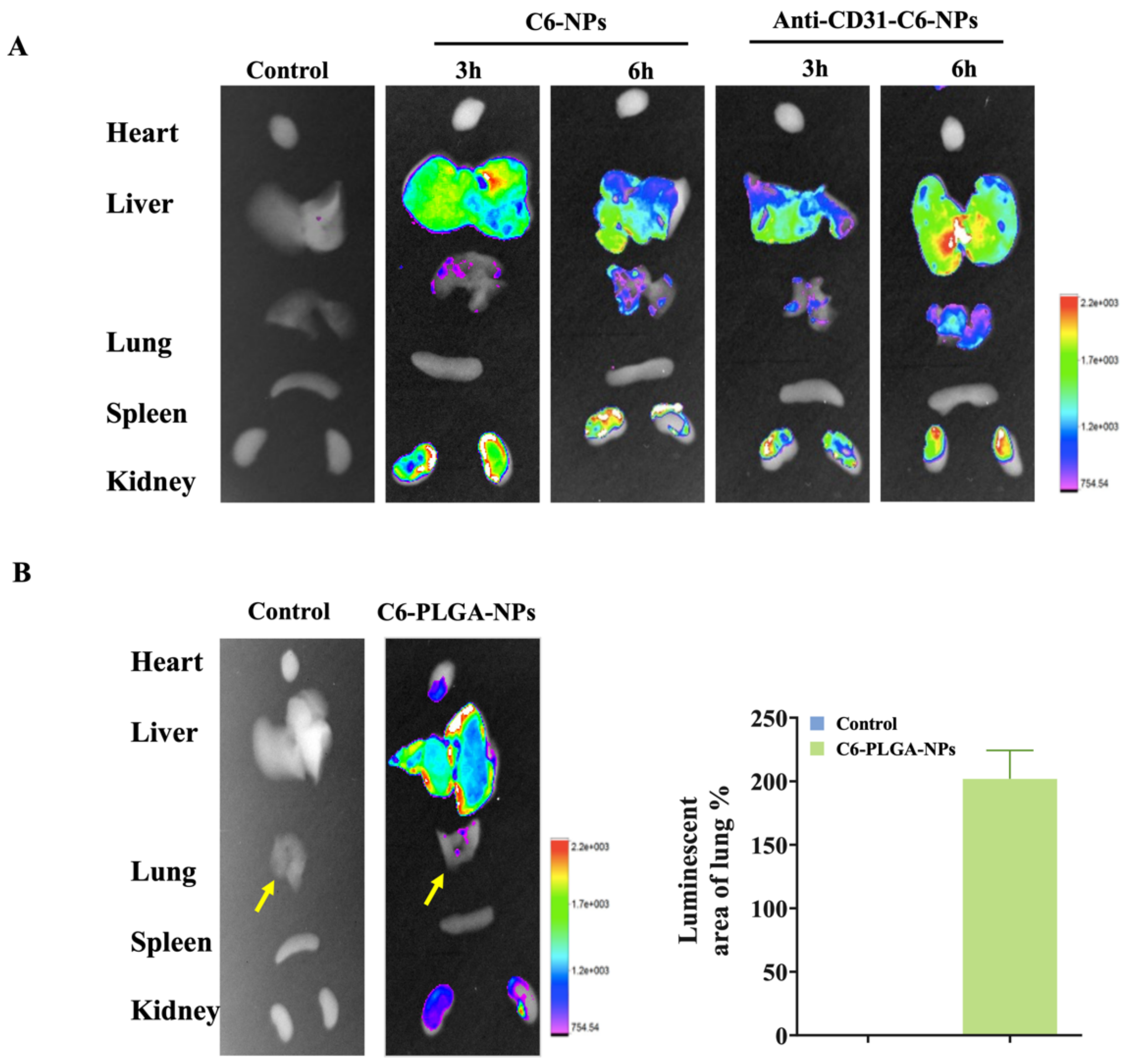

Supplementary Figure 5 (A)C57BL/6 mice (n=3) were intravenously injected with 5 mg kg^{-1} NPs (Control) C6-NPs and anti-CD31-C6-NPs, respectively. The organs including heart, liver, lung, spleen and kidney were collected for fluorescence analysis by IVIS after 3 and 6 hours. The NPs biodistribution was analyzed by *in vivo* imaging system to calculate the fluorescence. (B) C57BL/6 mice (n=3) were intravenously injected with 5 mg kg^{-1} NPs (Control) and C6-PLGA NPs, respectively. The organs including heart, liver, lung, spleen and kidney were collected for fluorescence analysis by IVIS after 6 hours.

Supplementary Figure. 6

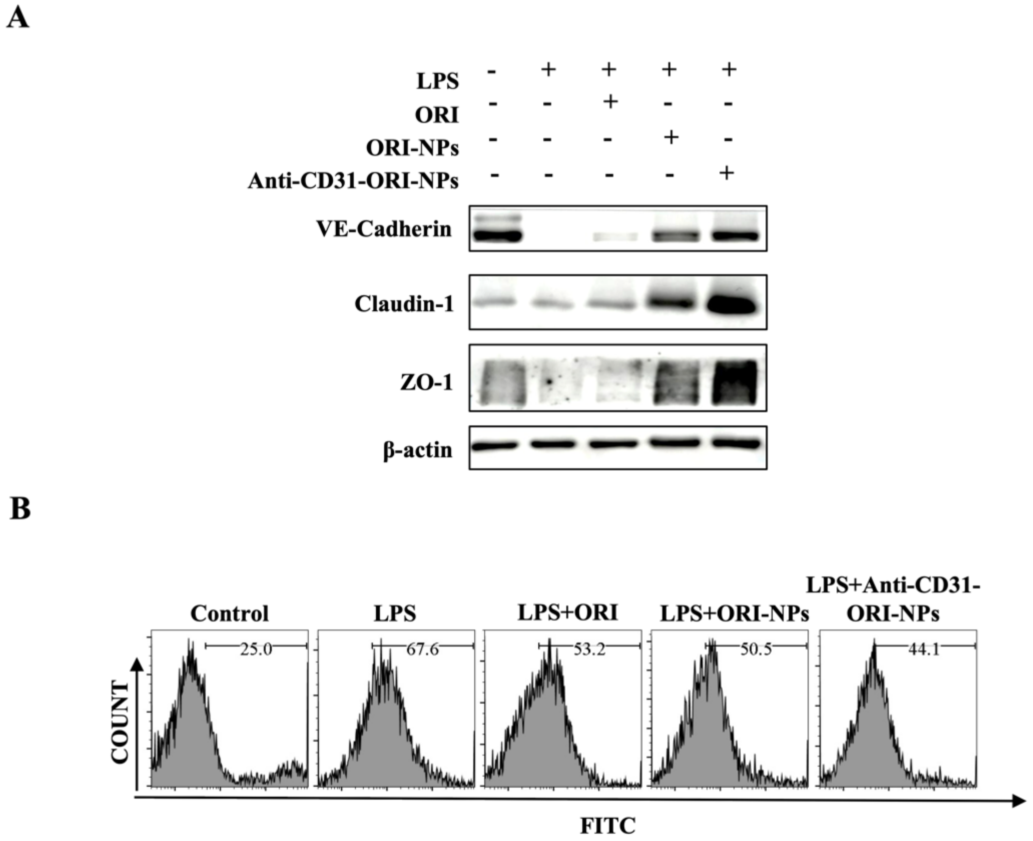

Supplementary Figure 6 Anti-CD31-ORI-NPs increases the anti-inflammatory effect. C57BL/6 mice (n=10) were pretreated with 5 mg kg^{-1} ORI, ORI-NPs and anti-CD31-ORI-NPs for 1 hour and then intratracheally injected with 2.5 mg kg^{-1} LPS. Mice were sacrificed and lungs were collected. (A) The expression of VE-Cadherin, Claudin-1, ZO-1 and β-actin in tissue were analyzed by western blot. Data were representative three independent experiments. (B) ROS production in the lung tissues was determined by flow cytometry. Data were representative three independent experiments.

Supplementary Figure. 7

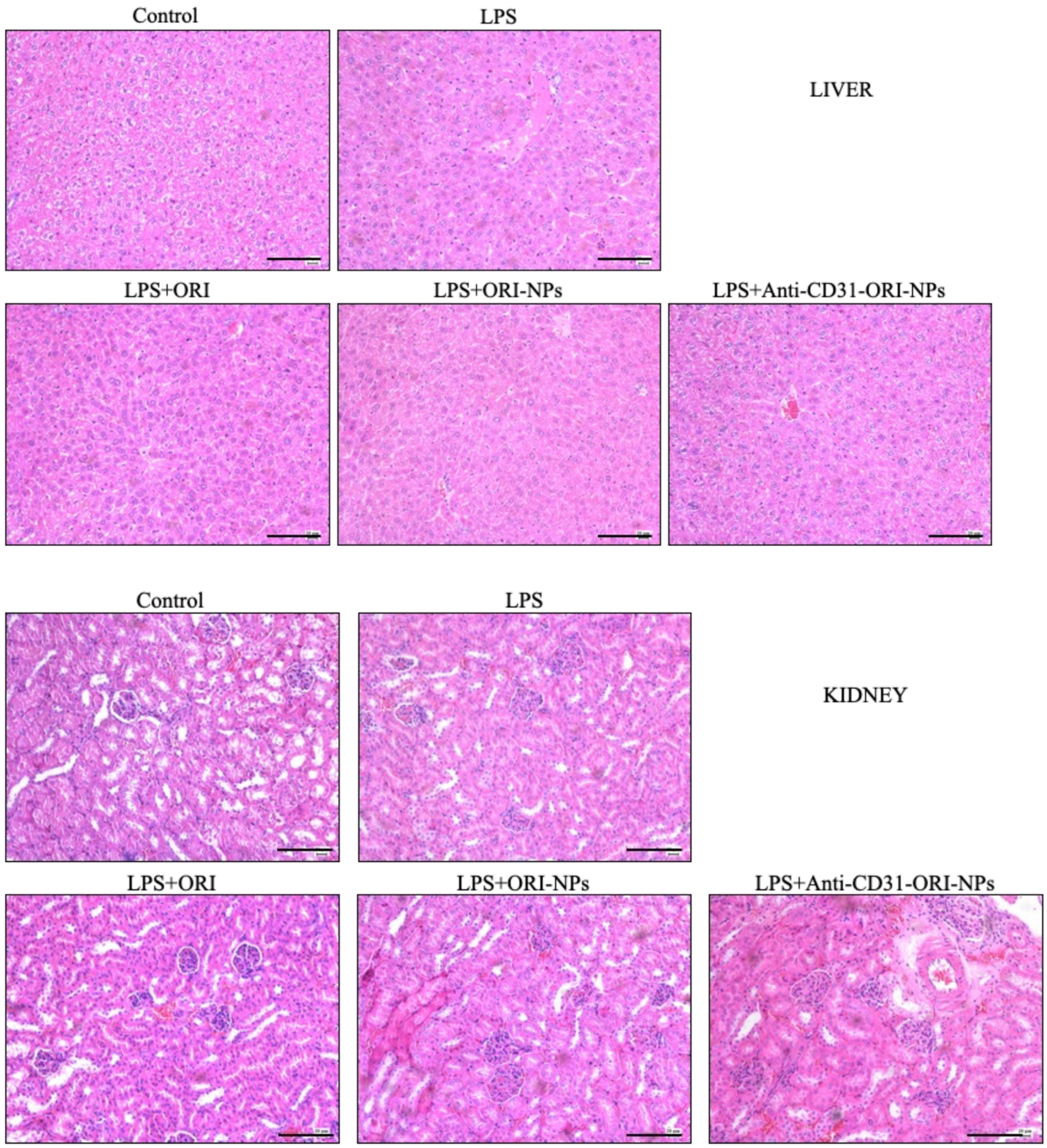

Supplementary Figure. 7 C57BL/6 mice (n=10) were intravenously injected 5 mg kg^{-1} NPs, ORI, ORI-NPs and anti-CD31-ORI-NPs for 1 hour, respectively. Mice were then intratracheally injected with 2.5 mg kg^{-1} LPS. Mice were sacrificed and the tissues were collected. Representative histological section of the livers and kidneys were stained with hematoxylin and eosin. Bars: 100 μm. Magnification × 100.
